# Young blood-induced rejuvenation of neurovascular coupling involves endothelial IGF-1/IGF-1R signaling: evidence from heterochronic parabiosis using endothelial IGF-1R deficient and systemic IGF-1 knockdown mice

**DOI:** 10.1007/s11357-026-02118-w

**Published:** 2026-02-20

**Authors:** Rafal Gulej, Roland Patai, Siva Sai Chandragiri, Mark Nagykaldi, Evelyn Brunner, Peter Mukli, Dorina Nagy, Kiana Vali Kordestan, Shoba Ekambaram, Santny Shanmugarama, Raghavendra Yelahanka Nagaraja, Andriy Yabluchanskiy, Stefano Tarantini, Anna Csiszar, Zoltan Ungvari

**Affiliations:** 1 Vascular Cognitive Impairment, Neurodegeneration, and Healthy Brain Aging Program, Department of Neurosurgery, University of Oklahoma Health Sciences Center, Oklahoma City, OK USA; 2 Oklahoma Center for Geroscience and Healthy Brain Aging, University of Oklahoma Health Sciences Center, Oklahoma City, OK USA; 3 International Training Program in Geroscience, Doctoral College, Health Sciences Division/Institute of Preventive Medicine and Public Health, Semmelweis University, Budapest, Hungary; 4 Institute of Translational Medicine, Semmelweis University, Budapest, Hungary; 5 Stephenson Cancer Center, University of Oklahoma, Oklahoma City, OK USA; 6 Healthy Aging Program, Institute for Translational Research, Budapest, Hungary

**Keywords:** Aging, Heterochronic parabiosis, Cell non-autonomous, Neuroendocrine, Neurovascular coupling, Cerebral microcirculation, Endothelial function, Insulin-like growth factor 1 (IGF-1), IGF-1 receptor (IGF-1R), Endothelial IGF-1R deficiency, Systemic IGF-1 knockdown, Vascular cognitive impairment and dementia, VCID, Cerebrovascular aging

## Abstract

Aging is accompanied by progressive impairment of neurovascular coupling (NVC), the mechanism that matches local cerebral blood flow to neuronal activity, contributing to cognitive decline and the development of vascular cognitive impairment and dementia. Exposure to a young systemic milieu through heterochronic parabiosis has been shown to restore NVC in aged mice, suggesting that circulating factors can rejuvenate cerebrovascular function. Yet, the molecular mediators responsible for this effect remain poorly defined. Building on our long-standing research demonstrating that age-related decline in insulin-like growth factor-1 (IGF-1) signaling contributes to cerebrovascular aging and NVC dysfunction, and on recent transcriptomic evidence implicating IGF-1 receptor (IGF-1R) activation in vascular rejuvenation, we hypothesized that the IGF-1/IGF-1R axis plays a role in the young blood-induced restoration of NVC. To test this, we combined heterochronic parabiosis with two complementary transgenic approaches: systemic IGF-1 knockdown (TBG-Cre-AAV8/Igf1^fl/fl^) and endothelial-specific IGF-1R deficiency (VE-Cadherin-Cre^ERT2^/Igf1r^fl/fl^). NVC responses to whisker stimulation were assessed in the somatosensory cortex using laser speckle contrast imaging. Exposure of aged wild-type parabionts to young circulation markedly improved NVC responses, confirming the rejuvenating effect of young blood. This improvement was significantly blunted in aged parabionts paired with IGF-1–deficient young partners and in endothelial IGF-1R–deficient aged parabionts exposed to young blood. These findings demonstrate that both circulating IGF-1 and endothelial IGF-1R signaling contribute to, but do not fully account for, the restoration of NVC in aged mice. Together, our results identify IGF-1/IGF-1R signaling as a critical component of the molecular network through which young blood exerts its neurovascular rejuvenating effects, while indicating that additional circulating and endothelial pathways also participate in the systemic regulation of cerebrovascular aging.

## Introduction

Aging is accompanied by a progressive decline in the function of the cerebral microcirculation, including impaired neurovascular coupling (NVC), which is the homeostatic mechanism that adjusts local cerebral blood flow to meet the metabolic demands of active neurons. This deterioration in NVC contributes to cognitive deficits and the pathogenesis of vascular cognitive impairment and dementia (VCID) [1–3]. VCID is increasingly prevalent in individuals over 65 years of age and represents the second most frequent cause of age-related cognitive impairment, whereas the pathogenesis of Alzheimer’s disease (AD) also involves microvascular pathologies and NVC dysfunction [4]. Given the projected threefold increase in global dementia burden by 2050 [5–8], understanding the microvascular mechanisms of cognitive decline has become an urgent priority.

Over the past two decades, our group and others have demonstrated that aging-related decline in insulin-like growth factor-1 (IGF-1) levels and signaling through its receptor (IGF-1R) plays a pivotal role in cerebromicrovascular aging [9–22]. Growth hormone (GH), secreted by the anterior pituitary in a pulsatile manner, is the principal endocrine regulator of hepatic IGF-1 production. Upon binding to GH receptors on hepatocytes, GH activates the JAK2/STAT5 signaling cascade, inducing transcription of the *Igf1* gene and systemic release of IGF-1. With advancing age, GH secretion declines markedly, a phenomenon often termed the “somatopause,” leading to a sustained reduction in plasma IGF-1 levels and consequently attenuated IGF-1/IGF-1R signaling in peripheral organs and the cerebral microvasculature [23]. Preclinical models demonstrate that deficiency in circulating IGF-1 leads to microvascular rarefaction, endothelial dysfunction, oxidative stress, and neurovascular uncoupling [10, 12, 17–19, 21, 22, 24–26]. Restoration of IGF-1 signaling, conversely, improves endothelial nitric oxide bioavailability, mitochondrial function, and NVC responses [18, 19, 21, 24]. Clinical studies similarly link low circulating IGF-1 levels to impaired NVC and cognitive decline in older adults [24]. Collectively, these studies have established the somatotropic/IGF-1 axis as a key regulator of cerebrovascular health and neurovascular function across the lifespan.

In parallel with this mechanistic line of investigation, recent discoveries in geroscience have highlighted that exposure to a young systemic milieu via heterochronic parabiosis can reverse multiple hallmarks of vascular and brain aging through cell-non-autonomous mechanisms [27–34]. Our previous studies demonstrated that aged mice exposed to young circulation (heterochronic parabiosis) exhibit significant restoration of NVC responses, improved blood–brain barrier (BBB) integrity, and enhanced capillarization [35, 36]. However, the molecular mediators of these rejuvenating effects remain unknown. Transcriptomic analysis of aortas from heterochronic aged parabionts revealed activation of IGF-1R–dependent signaling pathways as a key feature of vascular rejuvenation [37].

The convergence of these two independent research trajectories: (i) the long-standing evidence implicating IGF-1/IGF-1R deficiency in cerebrovascular and NVC decline, and (ii) the transcriptomic discovery of IGF-1R activation during parabiosis-induced vascular rejuvenation, led us to hypothesize that the IGF-1/IGF-1R signaling axis contributes to the neurovascular rejuvenation induced by young blood. To directly test this hypothesis, we employed a complementary experimental strategy that integrates heterochronic parabiosis with genetic models targeting distinct components of the IGF-1 signaling pathway. Specifically, we used endothelial-specific IGF-1R–deficient mice (VE-Cadherin-Cre^ERT2^/Igf1r^fl/fl^) to determine whether endothelial responsiveness to circulating IGF-1 is required for neurovascular rejuvenation, and systemic IGF-1–deficient mice (TBG-Cre/AAV8-Igf1^fl/fl^) to assess the role of endocrine IGF-1 availability in mediating the beneficial effects of young circulation. Neurovascular coupling responses were quantified in aged parabionts under each condition using laser speckle contrast imaging. This dual-transgenic, cross-circulation design allowed us to dissect the contribution of systemic and endothelial IGF-1/IGF-1R signaling to the restoration of cerebrovascular function in aging and to determine whether these mechanisms act in concert with additional circulating rejuvenating factors.

## Materials and methods

### Transgenic mouse models and animal husbandry

To generate a model of endothelial IGF-1R deficiency, Igf1r^fl/fl^ mice (B6;129-Igf1r^tm2Arge^/J; loxP sites flanking exon 3, Jackson Laboratory) were crossed with VE-Cadherin-Cre^ERT2^ mice (C57BL/6-Tg(Cdh5-Cre^ERT2^)1Rha; Taconic Biosciences). At 3 months of age, offspring received five consecutive daily intraperitoneal injections of tamoxifen (75 mg/kg body weight, dissolved in corn oil) to induce Cre-mediated, endothelial-specific deletion of *Igf1r*. Control littermates received vehicle (corn oil) injections.

For systemic IGF-1 knockdown, male homozygous Igf1^fl/fl^ mice (B6.129(FVB)-Igf1^tm1Dlr^/J; loxP sites flanking exon 4; Jackson Laboratory, backcrossed to the C57BL/6 background) were used. Hepatocyte-specific deletion of *Igf1* was achieved using adeno-associated virus serotype 8 (AAV8) vectors encoding Cre recombinase under the thyroxine-binding globulin (TBG) promoter (AAV8.TBG.PI.Cre.rBG) or control vectors expressing eGFP (AAV8.TBG.PI.eGFP.WPRE.bGH; AddGene, MA, USA). At approximately 3.5 months of age, mice were anesthetized with isoflurane and injected retro-orbitally with 1.3 × 10^10^ viral genomes diluted in 100 µL sterile 0.9% saline. Cre-mediated excision in these mice yields a truncated, non-functional IGF-1 protein incapable of receptor binding, resulting in a liver-specific reduction of circulating IGF-1 and decreased systemic IGF-1 signaling. This model has been previously validated to produce robust, hepatocyte-specific suppression of circulating IGF-1 without affecting extrahepatic IGF-1 expression and has been extensively characterized in prior studies from our group [20, 21, 38].

All experiments were performed using male mice exclusively, in order to minimize variability related to sex hormones and estrous cycling, and to ensure consistency with our prior studies investigating IGF-1 signaling, heterochronic parabiosis, and neurovascular aging.

All animals were housed in the Rodent Barrier Facility at the University of Oklahoma Health Sciences Center (OUHSC) under specific pathogen-free conditions, maintained on a 12-h light/dark cycle, and provided ad libitum access to water and a standard AIN-93G diet. Wild-type C57BL/6 mice (from the NIA colony maintained by Charles River Laboratories) served as parabiotic partners for the transgenic mice.

Parabiosis surgeries were performed using young (4.5-month-old) and aged (18-month-old) mice to establish four experimental groups: (1) aged endothelial IGF-1R–deficient parabionts paired with young wild-type partners (A_IGF1R-KD_-(Y)); (2) aged wild-type parabionts paired with IGF-1–deficient young partners (A-(Y_IGF1-KD_)); (3) wild-type heterochronic aged parabionts (A-(Y)); and (4) wild-type isochronic aged parabionts (A-(A)). Parentheses denote the non-measured partner in each pairing. Wild-type parabionts were not generated in the present study; instead, control data for young reference parabionts were obtained from a parallel cohort examined under identical experimental and analytical conditions [36], including anesthesia depth, whisker stimulation protocol, laser speckle contrast imaging system, data acquisition parameters, and experimenter, ensuring direct comparability between cohorts.

Following surgery, animals were returned to clean cages and received comprehensive postoperative care. Mice were monitored daily by research staff and veterinary personnel from the OUHSC Division of Comparative Medicine. All experimental procedures, including housing, surgery, intravital imaging, euthanasia, and tissue collection, were approved by the OUHSC Institutional Animal Care and Use Committee (protocols #21–031 and #24–021).

### Parabiosis surgery

Parabiosis surgeries were conducted between the following pairs: young and aged wild-type mice; aged wild-type and aged wild-type; aged VE-Cadherin-Cre^ERT2^/Igf1r^fl/fl^ and young wild-type mice; and aged wild-type and young TBG-Cre/Igf1^fl/fl^ mice, as previously described [35, 36].

Prior to surgery, candidate parabionts were co-housed for 5 days to facilitate social habituation. On the day of surgery, animals were anesthetized with isoflurane (3% in medical air, 0.6 L/min flow) and positioned on sterile drapes under separate vaporizers to allow independent control of anesthesia depth. The lateral sides of both mice were shaved and disinfected with alternating applications of 70% ethanol and povidone–iodine. Longitudinal skin incisions were made from the knee to the neck on corresponding sides of both animals. Opposing femurs and scapulae were bluntly exposed and sutured together to prevent separation, after which the skin edges were joined using continuous sutures.

During the procedure, mice received buprenorphine, meloxicam, warm saline, and enrofloxacin to provide analgesia, anti-inflammatory support, hydration, and infection prophylaxis. Pharmacological treatment and monitoring twice a day continued for 2 weeks postoperatively. Animals were monitored once daily thereafter for changes in body condition, wound integrity, hydration, and overall health until the experimental endpoint.

### Neurovascular coupling measurements

Neurovascular coupling responses were measured in ventilated parabionts using laser speckle contrast imaging (LSCI), as previously described [36]. Parabionts were anesthetized with isoflurane and intubated via intratracheal insertion of a 20G catheter. The skull of the aged partner was exposed by excising the overlying skin to permit imaging of speckle contrast changes corresponding to cerebral blood flow (CBF). Under continuous anesthesia, parabionts were positioned in ventral recumbency and transferred to the LSCI platform. Respiration and body temperature were continuously monitored and maintained using a SomnoSuite system (Kent Scientific Co.). NVC responses were recorded at 0.8–1.2% isoflurane in medical air (0.8 L/min flow) while maintaining body temperature between 36.0 and 37.0 °C.

Whisker stimulation was applied unilaterally for 30–45 s by continuous brushing with a cotton swab. CBF changes were quantified as the percentage change from baseline (30 s) in the contralateral somatosensory cortex. For each aged parabiont, at least 12 paired recordings of baseline and stimulation periods were obtained, and the mean response amplitude was calculated.

### Statistical analysis

All statistical analyses and graph preparation were performed using GraphPad Prism (version 10.1.0; GraphPad Software, San Diego, CA, USA). Data were analyzed using one-way ANOVA followed by Sidak’s post hoc test. Outliers were identified using Grubbs’ test (*α* = 0.05); one data point was excluded on this basis. Each experimental group included 7–8 biological replicates. Results are presented as mean ± standard deviation (SD). Statistical significance was accepted at *p < 0.05, **p < 0.001, ***p < 0.0001.

## Results

### Young blood–mediated restoration of NVC responses is attenuated in endothelial IGF-1R–deficient and systemic IGF-1–deficient heterochronic aged parabionts

Our previous work demonstrated that systemic, cell-non-autonomous mechanisms contribute to age-related alterations in neurovascular coupling [36]. However, the molecular mediators responsible for the rejuvenation of NVC in heterochronic aged parabionts remained unknown. Given that age-associated downregulation of insulin-like growth factor-1 and its receptor has been mechanistically linked to microvascular dysfunction and neurovascular uncoupling [18, 19, 21], we examined whether this signaling pathway contributes to the beneficial cerebrovascular effects of young blood.

**Fig. 1:**
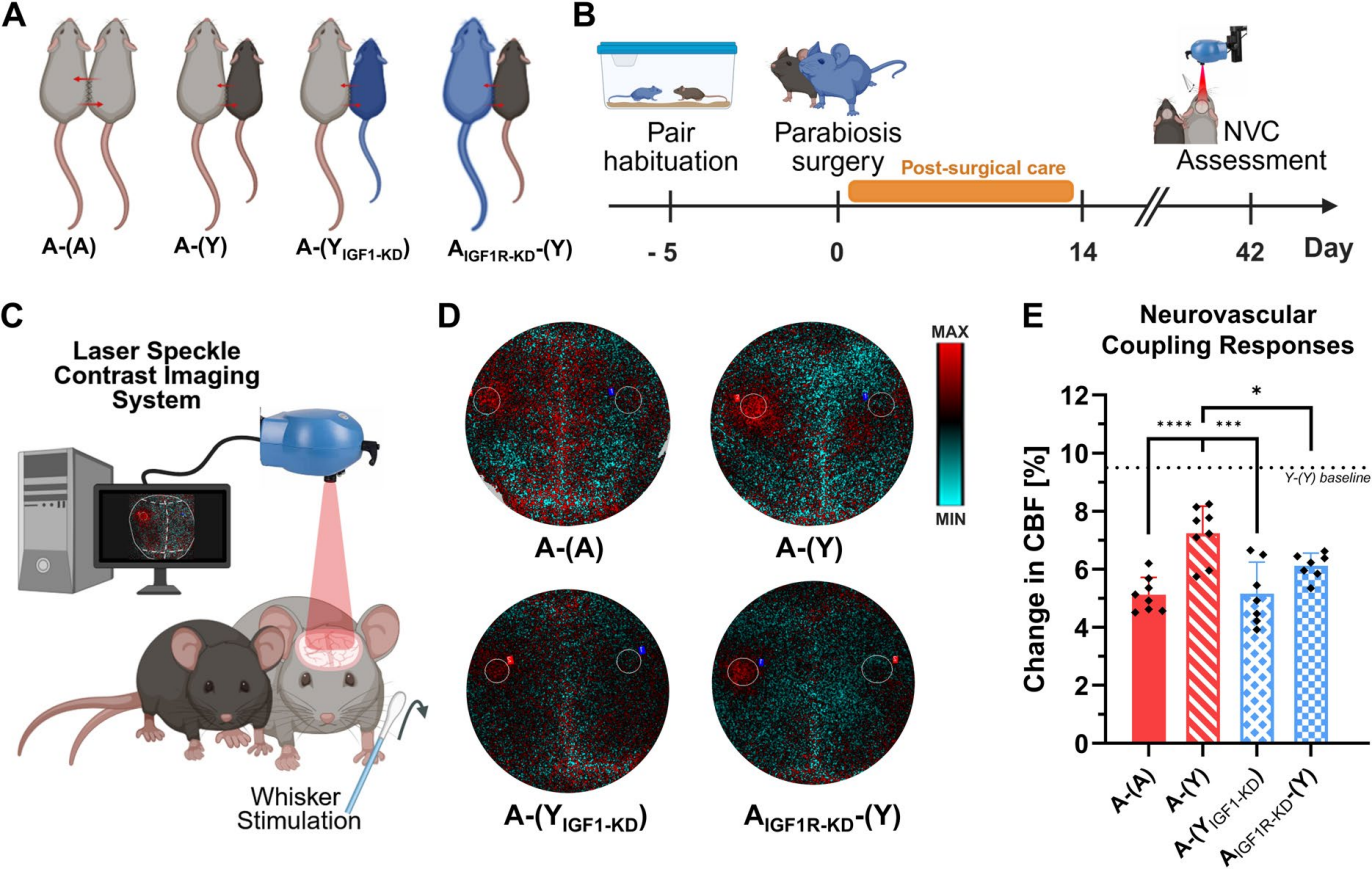
IGF-1/IGF-1R signaling contributes to young blood–mediated rejuvenation of cerebrovascular endothelial function and neurovascular coupling. **A** Experimental groups included wild-type aged isochronic parabionts (A–(A)), wild-type heterochronic aged parabionts (A–(Y)), aged parabionts paired with young IGF-1–deficient mice (A–(Y_IGF1-KD_)), and endothelial IGF-1R–deficient heterochronic aged parabionts (A_IGF1R-KD_–(Y)). Parentheses denote the non-measured parabiont in each pair. **B** Experimental timeline. **C** Schematic of whisker stimulation–induced NVC assessment using laser speckle contrast imaging in parabionts. **D** Representative differential maps showing changes in cerebral blood flow within the contralateral somatosensory cortex during whisker stimulation. Warmer colors indicate greater increases in CBF relative to baseline. **E** Quantification and statistical comparison of NVC responses across experimental groups. NVC data for wild-type parabionts (A–(A) and A–(Y)) were reported previously [36]. Data are shown as mean ± SD; *N* = 7–8 per group. Statistical analysis was performed using one-way ANOVA with Sidak’s post hoc test; *p < 0.05, ***p < 0.001, ****p < 0.0001

To test this hypothesis, we combined heterochronic parabiosis with complementary transgenic mouse models targeting either endothelial IGF-1R deficiency (A_IGF1R-KD_-(Y)) or systemic IGF-1 knockdown (A-(Y_IGF1-KD_)), and compared their NVC responses with wild-type heterochronic (A-(Y)) and isochronic aged (A-(A)) parabionts (Fig. 1A). NVC was assessed by laser speckle contrast imaging to visualize whisker stimulation–induced changes in cerebral blood flow in the contralateral somatosensory cortex (Fig. 1C,.

Consistent with our prior findings [36], aged mice exposed to young circulation (A-(Y)) exhibited a robust restoration of NVC responses compared with aged isochronic controls (A-(A); *p* < 0.001; Fig. 1E). In contrast, exposure to aged circulation in young parabionts (Y-(A)) significantly diminished NVC amplitude relative to young isochronic pairs (*p* < 0.01; data not shown), confirming that circulating factors in aged blood exert deleterious effects on cerebrovascular function. Thus, the NVC phenotype is bidirectionally regulated by systemic age, underscoring the critical role of circulating milieu in maintaining cerebrovascular homeostasis.

To determine the contribution of IGF-1/IGF-1R signaling to this systemic regulation, we next examined the transgenic cohorts. Representative LSCI difference maps revealed markedly reduced CBF increases in both A_IGF1R-KD_-(Y) and A-(Y_IGF1-KD_) parabionts compared with wild-type heterochronic aged pairs. Quantitative analysis confirmed that NVC responses were significantly attenuated in A_IGF1R-KD_-(Y) (*p* < 0.05) and A-(Y_IGF1-KD_) (*p* < 0.001) groups relative to A-(Y) controls (Fig. 1E). These data indicate that both endothelial IGF-1R signaling and circulating IGF-1 availability are required for the full cerebrovascular rejuvenating effect of young blood.

Interestingly, NVC responses in A_IGF1R-KD_-(Y) parabionts remained significantly higher than in their isochronic aged counterparts (A_IGF1R-KD_-(A); *p* < 0.05; data not shown), suggesting that while the IGF-1/IGF-1R axis is critical for optimal restoration, other circulating rejuvenating factors may partially compensate when this pathway is compromised.

Collectively, these results demonstrate that the IGF-1/IGF-1R signaling axis plays a necessary but not exclusive role in young blood–mediated restoration of neurovascular coupling, while aged blood exerts reciprocal detrimental effects on cerebrovascular function. These findings reinforce the concept that systemic age and endocrine–endothelial signaling interactions are major determinants of neurovascular health.

## Discussion

The present study demonstrates that exposure to a young systemic milieu, rich in circulating insulin-like growth factor 1, rejuvenates neurovascular coupling responses in aged mice, and that this effect is, at least in part, dependent on intact endothelial IGF-1/IGF-1R signaling. By combining heterochronic parabiosis with complementary transgenic approaches targeting either endothelial IGF-1R or systemic IGF-1 production, we provide direct mechanistic evidence that the somatotropic axis is a critical component of the young blood–mediated restoration of neurovascular function in aging. When either circulating IGF-1 levels in the young parabiont or endothelial IGF-1R expression in the aged parabiont were reduced, the NVC-enhancing effect of young blood was markedly attenuated, underscoring the importance of endocrine–endothelial communication in mediating these rejuvenating effects. These findings identify the IGF-1/IGF-1R pathway as a key, though not exclusive, element of the molecular network through which the systemic environment regulates neurovascular function across the lifespan.

Declining growth hormone and IGF-1 levels represent one of the most consistent endocrine changes during aging. This “somatopause” contributes to progressive structural and functional deterioration of the microvasculature, including endothelial senescence, mitochondrial dysfunction, increased production of reactive oxygen species, and impaired nitric oxide (NO)–mediated vasodilation [10, 12, 17–19, 21, 22, 24–26]. Our findings extend this concept by showing that restoration of circulating IGF-1 and intact endothelial IGF-1R responsiveness is necessary for full rejuvenation of NVC in aged brains. IGF-1/IGF-1R signaling promotes endothelial nitric oxide synthase (eNOS) activation through the PI3K/Akt pathway, enhances mitochondrial efficiency, and reduces oxidative stress [39, 40]—mechanisms that together maintain the bioavailability of NO and sustain the fine-tuned neurovascular responses required for functional hyperemia. The observation that partial restoration occurs even when endothelial IGF-1 signaling is compromised implies that the systemic milieu acts through redundant or convergent mechanisms, where IGF-1 represents one critical node within a broader rejuvenation network.

Endothelial cells occupy a central position in the neurovascular unit (NVU). They not only release vasodilator NO in response to neuronal and astrocytic cues but also propagate upstream vasodilatory signals via gap-junctional conduction from capillaries to resistance arterioles [41]. IGF-1/IGF-1R signaling modulates these processes by promoting eNOS activation, stabilizing mitochondria, limiting ROS generation, and maintaining intercellular junctional integrity [12, 13, 40]. The blunted NVC improvement observed in endothelial IGF-1R–deficient aged parabionts suggests that endothelial sensitivity to circulating IGF-1 is essential for translating systemic rejuvenating cues into local hemodynamic responses.

Importantly, our previous work also demonstrated that astrocytes express functional IGF-1 receptors and that astrocyte-specific IGF-1R knockdown impairs NVC in young mice, mimicking the aging phenotype [18]. Together, these data support a model in which both endothelial and astrocytic IGF-1R signaling act as effector sites through which circulating IGF-1 maintains NVU integrity and cerebrovascular reactivity. In aging, reduced IGF-1 bioavailability and receptor signaling weaken this multicellular communication network, promoting neurovascular uncoupling, cerebral hypoperfusion, and cognitive decline.

Our findings also confirm previous observations that exposure to aged circulation impairs NVC even in young mice, indicating that circulating pro-geronic factors can suppress neurovascular responsiveness. Among these, senescent cells and their senescence-associated secretory phenotype (SASP) represent a plausible contributor to the detrimental effects of aged circulation [42]. SASP factors, including pro-inflammatory cytokines and chemokines, can impair endothelial function, antagonize IGF-1/IGF-1R signaling, and disrupt neurovascular communication [42]. The reciprocal effects—enhancement of NVC in aged parabionts exposed to young blood and suppression in young parabionts exposed to aged blood—underscore that pro-geronic and anti-geronic factors in the systemic milieu dynamically modulate cerebrovascular function [35, 36]. These results are consistent with earlier studies showing that young plasma or therapeutic plasma exchange can transiently improve endothelial function, whereas aged plasma promotes oxidative stress and inflammation [43−45]. Collectively, these data reinforce that cerebrovascular aging is not purely cell-autonomous but systemically regulated, opening avenues to target endocrine and metabolic pathways to preserve vascular–brain communication and maintain a youthful microcirculatory phenotype.

Although our findings establish a causal link between IGF-1/IGF-1R signaling and young blood–induced NVC rejuvenation, the incomplete loss of benefit in IGF-1/IGF-1R–deficient mice indicates that additional circulating pathways contribute. Transcriptomic and proteomic studies have identified several candidate rejuvenating mediators, including GDF11, TIMP2, oxytocin, NAD⁺/SIRT1, and platelet factor 4, that may complement or intersect with IGF-1 signaling [32, 33, 46–53]. Conversely, age-associated pro-geronic mediators such as β₂-microglobulin and pro-inflammatory cytokines may antagonize these effects [27, 54–60]. The net cerebrovascular phenotype therefore reflects the balance between youthful protective and aged detrimental circulating factors, with IGF-1 signaling forming a central axis of this multifactorial rejuvenation network.

While these findings reveal a mechanistic contribution of IGF-1/IGF-1R signaling to the rejuvenation of NVC by young blood, therapeutic plasma or blood transfusion is not a viable rejuvenation strategy. Besides obvious logistical and ethical constraints, plasma-based interventions provide only transient changes in systemic composition and carry risks of immune sensitization, infection, and hemodynamic stress. More importantly, young blood contains hundreds of pleiotropic factors whose uncontrolled introduction could disrupt homeostasis. A rational translational approach should instead aim to mimic specific signaling pathways, for example, by restoring IGF-1 bioavailability, enhancing endothelial IGF-1R responsiveness, or modulating downstream eNOS/mitochondrial pathways, while minimizing systemic side effects. Conversely, excessive suppression of GH/IGF-1 signaling, as proposed in certain longevity paradigms, may paradoxically accelerate microvascular and cognitive decline.

The heterochronic parabiosis model, though powerful, also carries inherent limitations, including surgical stress, shared organ systems, and broad systemic exposure that complicates identification of specific mediators. Nevertheless, by combining parabiosis with transgenic dissection of IGF-1 signaling, our study provides strong causal evidence linking the somatotropic axis to cerebrovascular rejuvenation. While the present study focused on male mice, future investigations will be necessary to determine whether sex-specific differences exist in the contribution of IGF-1/IGF-1R signaling to neurovascular aging and rejuvenation, particularly in light of reported sex-dependent effects of IGF-1R modulation on lifespan and metabolism [61].

In summary, our findings demonstrate that IGF-1/IGF-1R signaling contributes to, but does not fully account for, the restoration of neurovascular coupling in aged mice exposed to young blood. The results highlight the pivotal role of endothelial IGF-1R signaling in maintaining neurovascular function and underscore that targeted modulation of the somatotropic axis may offer a promising strategy to preserve cerebrovascular health and cognitive resilience during aging.

## Data Availability

The datasets generated and analyzed during the present study are not publicly available due to privacy considerations. However, anonymized data may be obtained from the corresponding author upon reasonable request and will be provided in compliance with institutional and ethical regulations.
